# Aberrant pattern of regional cerebral blood flow in Alzheimer's disease: a voxel-wise meta-analysis of arterial spin labeling MR imaging studies

**DOI:** 10.18632/oncotarget.21475

**Published:** 2017-10-04

**Authors:** Hai Rong Ma, Ping Lei Pan, Li Qin Sheng, Zhen Yu Dai, Gen Di Wang, Rong Luo, Jia Hui Chen, Pei Rong Xiao, Jian Guo Zhong, Hai Cun Shi

**Affiliations:** ^1^ Department of Neurology, Traditional Chinese Medicine Hospital of Kunshan, Kunshan, PR China; ^2^ Department of Neurology, Affiliated Yancheng Hospital, School of Medicine, Southeast University, Yancheng, PR China; ^3^ Department of Radiology, Affiliated Yancheng Hospital, School of Medicine, Southeast University, Yancheng, PR China

**Keywords:** Alzheimer’s disease, arterial spin labeling, default mode network, meta-analysis, seed-based d mapping

## Abstract

Many studies have applied arterial spin labeling (ASL) to characterize cerebral perfusion patterns of Alzheimer's disease (AD). However, findings across studies are not conclusive. A quantitatively voxel-wise meta-analysis to pool the resting-state ASL studies that measure regional cerebral blood flow (rCBF) alterations in AD was conducted to identify the most consistent and replicable perfusion pattern using seed-based d mapping. The meta-analysis, including 17 ASL studies encompassing 327 AD patients and 357 healthy controls, demonstrated that decreased rCBF in AD patients relative to healthy controls were consistently identified in the bilateral posterior cingulate cortices (PCC)/precuneus, bilateral inferior parietal lobules (IPLs), and left dorsolateral prefrontal cortex. The meta-regression analysis showed that more severe cognitive impairment in the AD samples correlated with greater decreases of rCBF in the bilateral PCC and left IPL. This study characterizes an aberrant ASL-rCBF perfusion pattern of AD involving the posterior default mode network and executive network, which are implicated in its pathophysiology and hold promise for developing imaging biomarkers.

## INTRODUCTION

Alzheimer's disease (AD), the most prevalent type of dementia in the aging population [1–3], is featured by memory disturbance, attentional and executive deficits, and visuospatial and perceptual impairments [4]. The neuropathological hallmark of AD is the progressive accumulation of amyloid beta (Aβ) plaques and tau-related neurofibrillary tangles, and eventually accompanied by the damage and death of neurons in the brain [5]. The underlying neurobiology is far from being complete and no effective medications are available today for AD to slow or halt the damage and destruction of neurons [2]. This disorder has caused a substantial burden not only on the patients and their caregivers but also on the socioeconomic system [6]. Understanding the neural basis and early detection of AD are therefore very important.

Arterial spin labeling (ASL) MR imaging is a noninvasive technique that can quantitatively measure cerebral blood flow (CBF) by magnetically labelling the inflowing arterial blood water *in vivo* as an endogenous tracer [7–9]. ASL allows an automated voxel-by-voxel statistical analysis for regional CBF (rCBF) differences without any priori hypothesis [10–12]. Regional CBF is recognized as a reflection of intrinsic neural activity and brain physiology, which has been validated in normal aging and neuropsychiatric disorders [13–15]. ^18^Fluorodeoxyglucose positron emission tomography (FDG-PET) imaging biomarkers have been proposed by the National Institute on Aging and the Alzheimer's Association for detecting AD [16]. Perfusion pattern measured by ASL in the AD brain is closely matched with the metabolism pattern measured using PET [10, 17–20]. In addition, ASL offers a similar diagnostic ability as PET in the detection of AD [18–21]. Owing to its ease of acquisition, non-invasiveness, non-radiation, and reliability, ASL is increasingly proposed as an alternative to PET and holds promise for developing imaging biomarkers [13, 15, 22].

In the last decade, a growing body of research has applied ASL to characterize cerebral perfusion patterns of AD [22]. Abnormalities of rCBF measured by restingstate ASL in patients with AD in comparison with healthy controls have been frequently reported in the temporoparietal and posterior cingulate regions [10, 17, 19, 23–36]. However, the detailed findings typically varied across studies. Abnormalities of rCBF in the frontal [10, 12, 17–19, 23, 29–31, 35, 37–40] and occipital regions [11, 27, 36, 41], basal ganglia [39, 42], thalamus [24, 41], and insula [24, 39, 42] have also been reported. Although majority of ASL studies in AD demonstrated hypoperfusion in the brain regions, some studies also showed hyperperfusion in several cerebral areas [11, 25, 39]. These inconsistences may be presumably attributed to the differences in sample sizes, heterogeneity in the demographic and clinical variables of the samples as well as the variations in the technical characteristics of image acquisition and analytical methodology.

Consequently, it would be of great interest for this study to conduct a timely meta-analysis of ASL studies to identify the most consistent and replicable perfusion pattern of AD. This voxel-wise meta-analysis utilized the anisotropic effect-size version of seed-based *d* mapping (SDM) software [43, 44], which has been extensively applied in previous meta-analyses of neuroimaging studies for neuropsychiatric disorders [44–49].

## RESULTS

### Included studies

**Figure 1:**
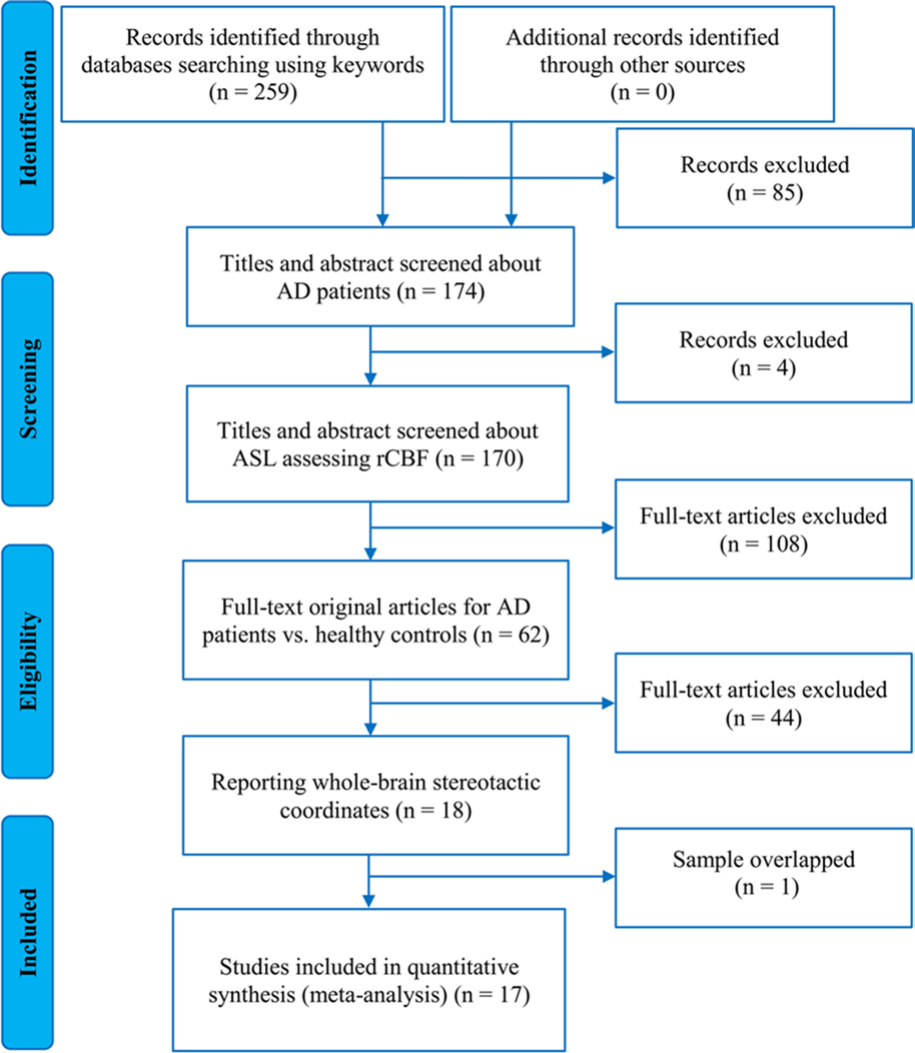
Flow diagram of the study selection procedure for the meta-analysis Abbreviations: AD, Alzheimer's disease; ASL, arterial spin labeling, rCBF, regional cerebral blood flow.

According to the strategy of literature search and study selection, a total of 17 ASL studies that investigated rCBF differences between 327 AD patients and 357 healthy controls were finally eligible for the meta-analysis [10, 11, 23–30, 37–42, 50]. Figure 1 presents a flow diagram of the studies that met the criteria for this meta-analysis. Of these included studies, thirteen were published in English and the other four were in Chinese [30, 37, 40, 41]. Fourteen out of the 17 studies were performed on the 3.0T MRI scanning systems and the other three were on the 1.5T MRI systems. Regarding the techniques applied to measure resting-state CBF in these studies, ten studies used pulsed ASL (PASL); five used pseudocontinuous ASL (pCASL); and the remaining two used continuous ASL (CASL). The quality score of each included study was no less than 8.5 (total score = 10), which indicates that the quality is acceptable. Table 1 summarizes the demographic, clinical, and technical characteristics as well as the quality scores of the ASL studies included in the meta-analysis. Supplementary Table 1 presents the diagnostic criteria, disease stage, cognitive test, and vascular burden assessment for AD of the included studies in the meta-analysis.

**Table 1:** Characteristics of ASL studies included in the meta-analysis

Study	Sample (female)		Mean Age (SD)	MMSE (SD)	Scanner Strength	Imaging technique	Software	FWHM	Threshold	Quality score#
Johnson et al., 2005	AD	20 (7)	72.9 (10.8)	21.0 (NA)	1.5 T	PASL	SPM99	12 mm	0.05, corrected	9.0
	HC	23 (13)	72.9 (8.2)	29.4 (NA)						
Alsop et al., 2008	AD	22 (12)	75.6 (9.2)	22.2 (5.9)	3.0 T	CASL	SPM2	8 mm	0.05, uncorrected	9.5
	HC	16 (9)	72.6 (8.9)	27.9 (2.6)						
Asllani et al., 2008	AD	12 (5)	70.7 (8.7)	38.7 (11.1)^*^	1.5 T	CASL	SPM99	6 mm	0.05, corrected	9.5
	HC	20 (12)	72.1 (6.5)	53.5 (2.8)^*^						
Yoshiura et al., 2009	AD	20 (10)	73.5 (9.6)	20.4 (4.3)	3.0 T	PASL	SPM2	12 mm	0.001, uncorrected	9.5
	HC	23 (12)	72.9 (6.7)	29.3 (0.9)						
Cantin et al., 2011	AD	9 (5)	71.1 (6.7)	21.7 (2.2)	1.5 T	PASL	SPM5	12 mm	0.05, corrected	8.5
	HC	11 (6)	65.4 (9.3)	29.5 (0.5)						
Chen et al., 2011	AD	15 (9)	72.0 (6.3)	19.9 (5.9)	3.0 T	pCASL	SPM5	12 mm	0.005, uncorrected	9.5
	HC	19 (12)	69.2 (7.6)	29.5 (1.0)						
Dashjamts et al., 2011	AD	23 (14)	74.6 (8.9)	21.1 (4.4)	3.0 T	PASL	SPM8	12 mm	0.001, uncorrected	9.5
	HC	23 (12)	73.2 (6.9)	29.4 (0.9)						
Alexopoulos et al., 2012	AD	19 (8)	72.0 (9.4)	NA	3.0 T	PASL	SPM5	12 mm	0.001, uncorrected	8.5
	HC	24 (16)	67.1 (6.1)	NA						
Mak et al., 2012	AD	20 (15)	75.4 (6.75)	16.3 (4.55)	3.0 T	PASL	SPM5	8 mm	0.001, uncorrected	9.0
	HC	20 (17)	70.8 (5.99)	28.5 (2.00)						
Grieder et al., 2013	AD	14 (NA)	66.5 (9.6)	24.8 (3.9)	3.0 T	pCASL	SPM8	8 mm	0.05, corrected	9.0
	HC	19 (NA)	69.5 (3.1)	28.7 (0.9)						
Kim et al., 2013	AD	25 (21)	70.9 (9.8)	17.2 (NA)	3.0 T	PASL	SPM5	12 mm	0.005, uncorrected	9.0
	HC	25 (16)	68.4 (5.6)	27.3(NA)						
Zhang and Fan, 2013	AD	16 (10)	76.00 (7.12)	19.25 (4.97)	3.0 T	pCASL	SPM8	NA	0.001, uncorrected	9.0
	HC	16 (12)	70.75 (7.95)	28.75 (0.93)						
Zhang et al., 2013	AD	17 (12)	66.92 (8.91)	15.92 (4.32)	3.0 T	PASL	SPM8	NA	0.05, corrected	9.0
	HC	17 (12)	66.07 (5.78)	28.00 (1.41)						
Ding et al., 2014	AD	24 (19)	74.58 (6.678)	16.0 (3.9)	3.0 T	pCASL	SPM8	6 mm	0.05, corrected	9.5
	HC	21 (13)	69.64 (5.884)	29.4 (1.0)						
Liu et al., 2014	AD	16 (10)	75.3 (6.9)	18.69 (5.50)	3.0 T	pCASL	SPM8	6 mm	0.05, corrected	9.5
	HC	19 (14)	69.7 (8.1)	28.84 (0.90)						
Lyu et al., 2015	AD	30 (15)	68 (10)	21.6 (1.6)	3.0 T	PASL	SPM8	8 mm	0.05, corrected	9.5
	HC	30 (19)	52 (8)	29.0 (1.0)						
Roquet et al., 2016	AD	25 (17)	73.6 (9.1)	19.5 (3.4)	3.0 T	PASL	SPM8	8 mm	0.05, corrected	9.5
	HC	21 (12)	64.8 (8.6)	28.9 (1.0)						

Abbreviations: ASL, arterial spin labeling, AD, Alzheimer's disease; HC, healthy controls; SD, standard deviation; MMSE, Mini-Mental State Examination; FWHM, full width at half maximum; NA, not available; PASL, pulsed arterial spin labeling; CASL, continuous arterial spin labeling; pCASL, pseudocontinuous arterial spin labeling; SPM, statistical parametric mapping; ^*^, modified MMSE; ^#^, a maximum score of 10 for each study.

### Regional CBF differences by pooling all included studies

**Figure 2:**
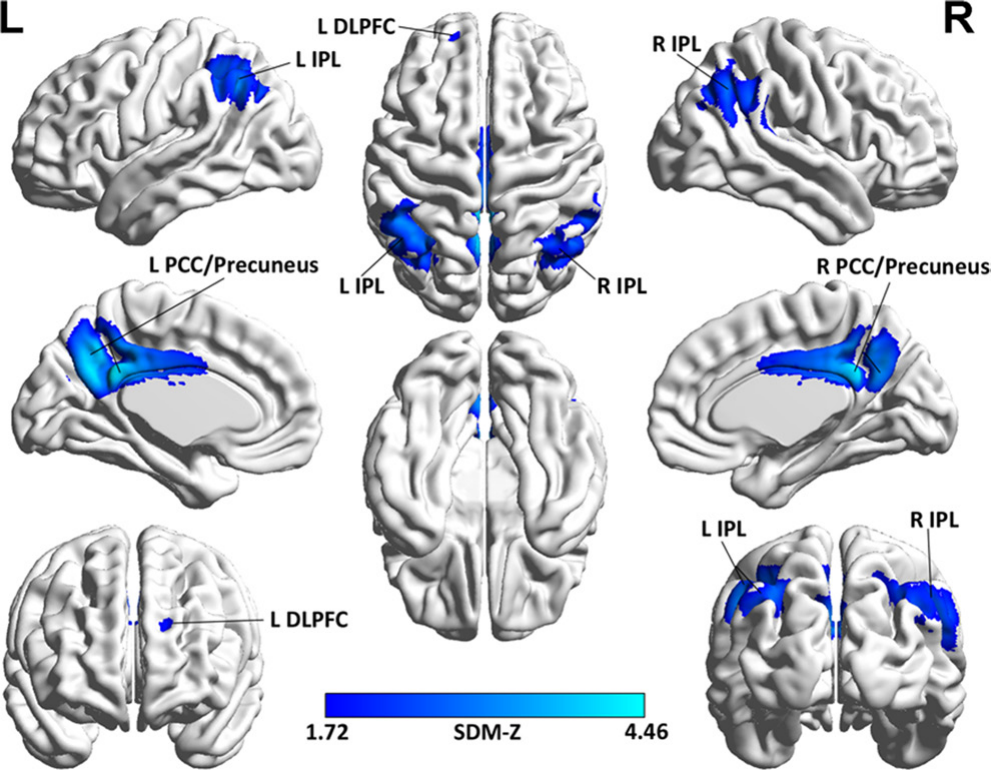
Brain map for the meta-analytic results of the 17 ASL studies comparing rCBF differences between AD patients and healthy controls Abbreviations: ASL, arterial spin labeling, rCBF, regional cerebral blood flow; AD, Alzheimer's disease; L, left; R, right, IPL, inferior parietal lobule; DLPFC, dorsolateral prefrontal cortex; PCC, posterior cingulate cortex; SDM, Seed-based *d* Mapping; The color bar indicates the maximum and the minimum absolute SDM-Z values.

The voxel-wise SDM analysis demonstrated that decreased rCBF in AD patients compared to healthy controls were mainly located in the bilateral posterior cingulate cortices (PCC)/precuneus, bilateral inferior parietal lobules (IPLs), and left dorsolateral prefrontal cortex (DLPFC). In contrast, no regions showed significant increases of rCBF in AD patients relative to healthy controls. The SDM results are described in Table 2 and illustrated in Figure 2.

**Table 2:** Clusters of regional CBF differences in patients with AD relative to healthy controls

	Anatomical label	Peak MNI coordinate (x, y, z)	Number of voxels	SDM-Z value	*p* value (SDM)	*p* value (Egger's test)
Decreased regional CBF	Bilateral PCC/precuneus (BAs 23, 7, and 26)	-2, -54, 30	4215	-4.46	∼0	0.051
	Left IPL (BAs 40, 39, and 7)	-50, -54, 40	2427	-3.37	∼0	0.15
	Right IPL (BAs 40, 39, and 7)	50, -60, 38	1161	-2.89	0.0000041	0.29
	Left DLPFC (BAs 9, 10, and 46)	-18, 52, 30	128	-1.89	0.0021	0.070
Increased regional CBF	None					

Abbreviations: CBF, cerebral blood flow; AD, Alzheimer's disease; MNI, Montreal Neurological Institute; SDM, Seed-based d Mapping; PCC, posterior cingulate cortex; IPL, inferior parietal lobule; DLPFC, dorsolateral prefrontal cortex; BA, Brodmann area.

### Reliability analysis

The whole-brain jackknife sensitivity analysis revealed that decreases of rCBF in the bilateral PCC/precuneus and bilateral IPLs were the most robust findings, replicable in all 17 combinations. Decreases of rCBF in the left DLPFC remained highly replicable, as it was preserved in all but four in combinations of studies (Table 3).

**Table 3:** Jackknife sensitivity analysis

All studies but …	Bilateral PCC/precuneus	Left IPL	Right IPL	Left DLPFC
Johnson et al., 2005	yes	yes	yes	no
Alsop et al., 2008	yes	yes	yes	yes
Asllani et al., 2008	yes	yes	yes	yes
Yoshiura et al., 2009	yes	yes	yes	yes
Cantin et al., 2011	yes	yes	yes	yes
Chen et al., 2011	yes	yes	yes	yes
Dashjamts et al., 2011	yes	yes	yes	yes
Alexopoulos et al., 2012	yes	yes	yes	yes
Mak et al., 2012	yes	yes	yes	no
Grieder et al., 2013	yes	yes	yes	yes
Kim et al., 2013	yes	yes	yes	yes
Zhang and Fan, 2013	yes	yes	yes	yes
Zhang et al., 2013	yes	yes	yes	yes
Ding et al., 2014	yes	yes	yes	yes
Liu et al., 2014	yes	yes	yes	no
Lyu et al., 2015	yes	yes	yes	yes
Roquet et al., 2016	yes	yes	yes	yes
**Total**	**17 out of 17**	**17 out of 17**	**17 out of 17**	**14 out of 17**

Abbreviations: PCC, posterior cingulate cortex; IPL, inferior parietal lobule; DLPFC, dorsolateral prefrontal cortex; yes, the region(s) reported; no, the region(s) not reported.

### Publication bias analysis

No publication biases for the identified brain regions with rCBF differences between AD patients and healthy controls were observed, which was revealed by the approximately symmetric funnel plots (Supplementary Figure 1) and the non-significant Egger's tests (Table 2).

### Meta-regression analysis

**Figure 3:**
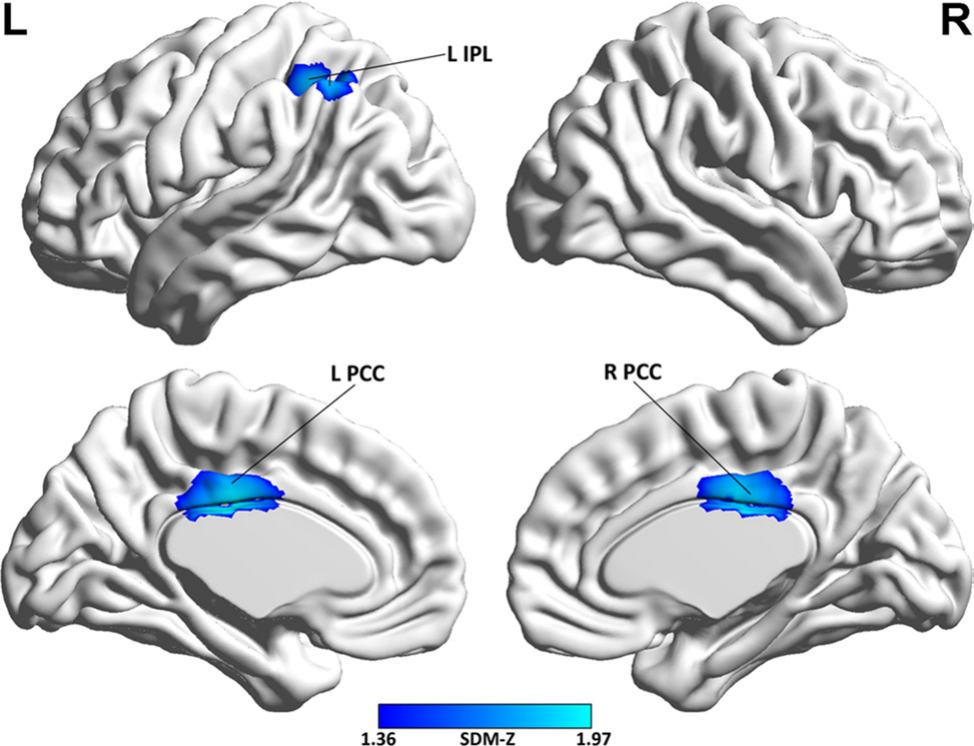
Brain map of the voxel-wise meta-regression analysis of MMSE scores in the AD samples Abbreviations: MMSE, Mini-Mental State Examination; AD, Alzheimer's disease; L, left; R, right, IPL, inferior parietal lobule; PCC, posterior cingulate cortex; SDM, Seed-based *d* Mapping; The color bar indicates the maximum and the minimum absolute SDM-Z values.

The meta-regression analysis showed that lower Mini-Mental State Examination (MMSE) scores in the AD samples were associated with greater decreases of rCBF in the bilateral PCC (Peak Montreal Neurological Institute (MNI) coordinate at x = −4, y = −22, z = 28; p = 0.0000036; SDM-Z = −1.97; 1149 voxels) and left IPL (Peak MNI coordinate at x = −38, y = −52, z = 50; p = 0.0000052; SDM-Z = −1.92; 761 voxels) (Figure 3).

## DISCUSSION

To the best of our knowledge, this is the first quantitative meta-analysis to pool the resting-state ASL studies to identify the most consistent pattern of rCBF and to explore its clinical correlation in AD. A total of 17 studies as measured with ASL in 327 AD patients and 357 healthy controls were included in this voxel-wise meta-analysis. Decreases of rCBF in AD patients relative to healthy controls are consistently identified in the bilateral PCC/precuneus, bilateral IPLs, and left DLPFC. Furthermore, the meta-regression analysis indicates that more severe cognitive impairment in the AD samples was associated with greater decreases of rCBF in the bilateral PCC and left IPL.

The bilateral PCC/precuneus and bilateral IPLs, where this study observed the most consistent brain regions of decreased rCBF in AD, are key nodes of the posterior default mode network (DMN) [51, 52]. The DMN, which is comprised of highly interconnected brain areas involved in higher cognitive functions, is the most active brain system at rest in healthy subjects [51]. There is compelling evidence suggesting that the DMN, especially the posterior part is functionally and structurally impaired in AD patients [53–59] and at-risk subjects [12, 59–61]. Dysfunction of the DMN, a hallmark of AD, has been linked to core memory, attentional, and visuospatial deficits [62, 63]. The DMN nodes, such as the PCC/precuneus, parietal and temporal lobes were confirmed to be more vulnerable to early amyloid deposition [54, 55, 64]. High amyloid-beta deposition in the DMN was demonstrated in AD patients [54, 65–68]. In addition, some studies suggest that decreases of rCBF in these regions, especially in the PCC/precuneus, may reflect a remote functional deficits caused by the neuronal damage in the medial temporal structures [10, 28, 69–72]. As such, decreases of rCBF in the bilateral PCC/precuneus and bilateral IPLs observed in this study may be a reflection of pathophysiological process that involves vascular dysfunction and neuronal degeneration underlying AD [14].

The perfusion pattern identified in the current meta-analysis is largely in line with the metabolism pattern from the voxel-wise meta-analyses of FDG-PET studies in AD patients [49, 73]. In addition, the perfusion pattern using ASL in AD are concordant with the perfusion pattern using other MRI modalities, such as H_2_^15^O-PET, ^99m^Tc-hexamethylpropyleneamine oxime (HMPAO)-single photon emission computed tomography (SPECT), and ^99m^Tc-ethyl cysteinate dimmer (ECD)-SPECT [73]. These data suggest that ASL provides information comparable to above invasive imaging techniques and shows potential as a reliable and safe alternative. The findings identified in the present voxel-wise meta-analysis are also consistent with the ASL studies using ROI analyses [10, 27, 34, 74, 75]. Decreases of rCBF in these areas could serve as specific regions of interest for a further diagnostic utility. Decreases of normalized rCBF in the bilateral PCC/precuneus had an accuracy of 86.0% (91.3% sensitivity and 80.0% specificity) in distinguishing AD patients from healthy controls [26]. Brain activity changes in the DMN may be an early marker for AD. Alexopoulos et al identified hypometabolism/hypoperfusion consistently in the DMN, especially the posterior part, such as the PCC/precuneus and IPLs in amnestic mild cognitive impairment (aMCI) converters [73]. They further demonstrated that hypometabolism/hypoperfusion in the IPLs was the most reliable predictor of the progression from aMCI to AD [73]. A recent study observed a continuing decrease of CBF in the PCC/precuneus and other related regions in the continuum of AD [76]. Moreover, our meta-regression analysis shows that the severity of cognitive impairment in the AD samples was associated with the rCBF changes in the bilateral PCC and left IPL, which suggests that altered rCBF in these regions may act as an imaging marker for tracking disease progression.

Besides the areas of rCBF changes observed in the DMN in AD patients compared with healthy controls, we also identified the regions of decreased rCBF belonging to the central executive network (CEN), such as the DLPFC and posterior parietal areas [77, 78]. The CEN is another intrinsic brain network that is known to be involved in executive functioning, particularly important for maintaining high-level cognition [78, 79]. Aberrant functional connectivity in the CEN was observed in AD [80–85]. Decreases of rCBF in the regions of the CEN probably account for the executive deficits in AD patients [79].

Atrophy of gray matter [86] and white matter [87] in the medial temporal lobe (MTL) is a characteristic and could serve as a neurostructural predictor of AD [88]. Interestingly, we did not observe consistent rCBF changes in MTL in AD patients relative to healthy controls in the present meta-analysis. This structural-functional discordance has been frequently detected in previous studies [10, 28, 69–71, 89], which is interpreted as a compensatory response to morphologic alterations [71]. Therefore, rCBF changes in the MTL may not be sensitive enough to distinguish healthy elders from AD patients, as aging-related tau pathology, hypometabolism, and hypoperfusion in the MTL were also observed in normal-aged individuals [68, 90–92].

### Limitations and future perspectives

Several limitations in this study should be acknowledged. First, a huge body of ASL studies in AD was excluded because of the chosen voxel-wise approach and this approach was based on summarized coordinates and their effect sizes rather than on raw imaging data or statistical brain maps, which might limit its accuracy [93]. In addition, the patients in the included studies were the clinical samples, who compared with community-based normal control volunteers. In this context, although they were matched or adjusted regarding age, sex ratio and education, some other critical factors, such as socioeconomic status, vascular risk burden, cognitive reserve, racial/ethnic make-up, and genetic vulnerability were not addressed in most of the original studies, which might lead to some heterogeneity in the conclusions and remains to be further addressed. Further analysis of multicenter raw imaging data in large homogeneous samples, like ASL-MRI scans from the Alzheimer's disease Neuroimaging Initiative (ADNI) subjects [94], would confirm the present findings. Second, our meta-analysis that synthesized the findings from the cross-sectional studies could not determine whether decreases of rCBF in the identified brain areas are the cause or consequence of AD [14]. Longitudinal studies could provide further insights. Third, ASL acquisition parameters, pre- and post-processing steps, such as scanner field-strength, inversion time, labeling duration, post label time delay, volume coverage, readout approaches, partial volume correction, and GM correction, may bias the results that warrant careful consideration by investigators. Further investigations in high field-strength MRI scanners with optimized and standardized imaging acquisition and analytical methodology are recommended [15].

## MATERIALS AND METHODS

### Literature search and study selection

A comprehensive literature search was performed in the PubMed, Web of Science, and Embase databases up until 16 December, 2016, using the keywords “Alzheimer's Disease” AND “arterial spin labeling”. The China National Knowledge Infrastructure (CNKI) database was searched for additional studies published in Chinese. Reference lists from relevant studies were manually reviewed for further eligible studies. Studies were included in the meta-analysis if they met the following criteria: 1) the study used the standard diagnostic criteria for Alzheimer's Disease [95–99]; 2) the study utilized resting-state ASL MR imaging to measure rCBF differences between Alzheimer's Disease and healthy controls; 3) the study applied a voxel-based statistical analysis; 4) the study reported three-dimensional coordinates in Talairach or MNI space; and 5) the study was published as an original article (not as a letter or a meeting abstract or a comment) in a peer-reviewed journal. Studies were excluded if they specifically used region of interest (ROI) approaches. Studies were also excluded if they did not report significant results with three-dimensional peak coordinates. To avoid duplication, only the study with a larger sample size was included in case that the patient populations overlapped. The quality of each eligible study was evaluated with a 10-point checklist (Supplementary Table 2) based on previous neuroimaging meta-analyses [100, 101]. This study followed the MOOSE guidelines for the meta-analyses of observational studies [102].

### Data analysis

#### Main voxel-wise meta-analysis

A meta-analysis of rCBF differences between AD patients and healthy controls was performed using the SDM software package (www.sdmproject.com). The SDM approaches have been described in detail previously [44–47, 93, 103–105]. We briefly summarized here. Peak coordinates and effect sizes (e.g., *t*-values) of rCBF differences between AD patients and healthy controls were firstly extracted from each study [103, 105]. The SDM software then separately recreated a standard MNI map of rCBF for each study applying an anisotropic Gaussian kernel (full width at half maximum [FWHM] = 20 mm) [44, 103, 105]. The mean map was generated by voxel-wise calculation of the mean of the study maps with a standard random-effects model, taking into account the sample size, the intra-study variability, and the between-study heterogeneity [44, 103, 105]. To generate significant results and the final map of rCBF, we applied the default SDM kernel size and threshold (uncorrected voxel *p* = 0.005, peak height Z = 1, cluster extent = 10 voxels), which is equivalent to a corrected *p* value of 0.05 and is found to optimally balance false positives and negatives [103, 105]. Results were visualized with the BrainNet Viewer [106].

### Reliability analysis

To test the reliability of the findings identified in the main voxel-wise meta-analysis, a whole-brain voxel-based jackknife sensitivity analysis was performed by iteratively repeating the same analysis, excluding one study at a time [44, 46, 93, 103].

### Analysis of publication bias

Possible publication bias was examined with a standard meta-analysis using the Stata 12.0 software (Stata Corp LP, College Station, TX, USA) by extracting the values from the relevant peaks from the main voxel-wise meta-analysis. An asymmetric funnel plot and a *p*-value less than 0.05 for Egger's test were considered significant.

### Meta-regression analysis

A meta-regression analysis was conducted to assess the severity of cognitive impairment examined by MMSE scores that correlate with the ASL results. A stringent threshold of *p* = 0.0005 and a cluster extent of 10 voxels were used for the meta-regression analysis [103, 104].

## CONCLUSIONS

Our study shows the most consistent and replicable decreases of rCBF in the bilateral PCC/precuneus, bilateral IPLs, and left DLPFC in AD patients compared with healthy controls via the voxel-wise meta-analysis of ASL studies. These aberrant regions predominantly involve in the default mode and central executive networks that are implicated in the AD pathophysiology. This study further demonstrates that reduced rCBF in the bilateral PCC/precuneus and left IPL was related to the severity of cognitive impairment in AD patients, which suggests that altered rCBF in these regions may act as an objective imaging marker for tracking AD progression.

## SUPPLEMENTARY MATERIALS FIGURE AND TABLES



## Abbreviations

AD: Alzheimer's disease
ADNI: Alzheimer's disease Neuroimaging Initiative
aMCI: amnestic mild cognitive impairment
ASL: arterial spin labeling
BA: Brodmann area
CASL: continuous arterial spin labeling
CBF: cerebral blood flow
CEN: central executive network
ECD-SPECT: 99mTc-ethyl cysteinate dimmer-single photon emission computed tomography
DLPFC: dorsolateral prefrontal cortex
DMN: default mode network
FDG-PET: ^18^Fluorodeoxyglucose positron emission tomography
FWHM: full width at half maximum
HC: healthy controls
HMPAO-SPECT: 99mTc-hexamethylpropyleneamine oxime-single photon emission computed tomography
IPL: inferior parietal lobule
MMSE: Mini-Mental State Examination
MNI: Montreal Neurological Institute
PCC: posterior cingulate cortex
pCASL: pseudocontinuous arterial spin labeling
PASL: pulsed arterial spin labeling
NA: not available
rCBF: regional cerebral blood flow
ROI: region of interest
SDM: Seed-based *d* Mapping
SD: standard deviation
SPM: statistical parametric mapping
CNKI: The China National Knowledge Infrastructure
