# Supplementary material for: Aberrant pattern of regional cerebral blood flow in Alzheimer's disease: a voxel-wise meta-analysis of arterial spin labeling MR imaging studies
Source: Oncotarget. 2017 Oct 4;8(54):93196–208. doi: 10.18632/oncotarget.21475 (PMC5696255; doi:10.18632/oncotarget.21475)
Supplement: Supplementary file 1 [file oncotarget-08-93196-s001.pdf]

Aberrant pattern of regional cerebral blood flow in Alzheimer's disease: a voxel-wise meta-analysis of arterial spin labeling MR imaging studies

SUPPLEMENTARY MATERIALS

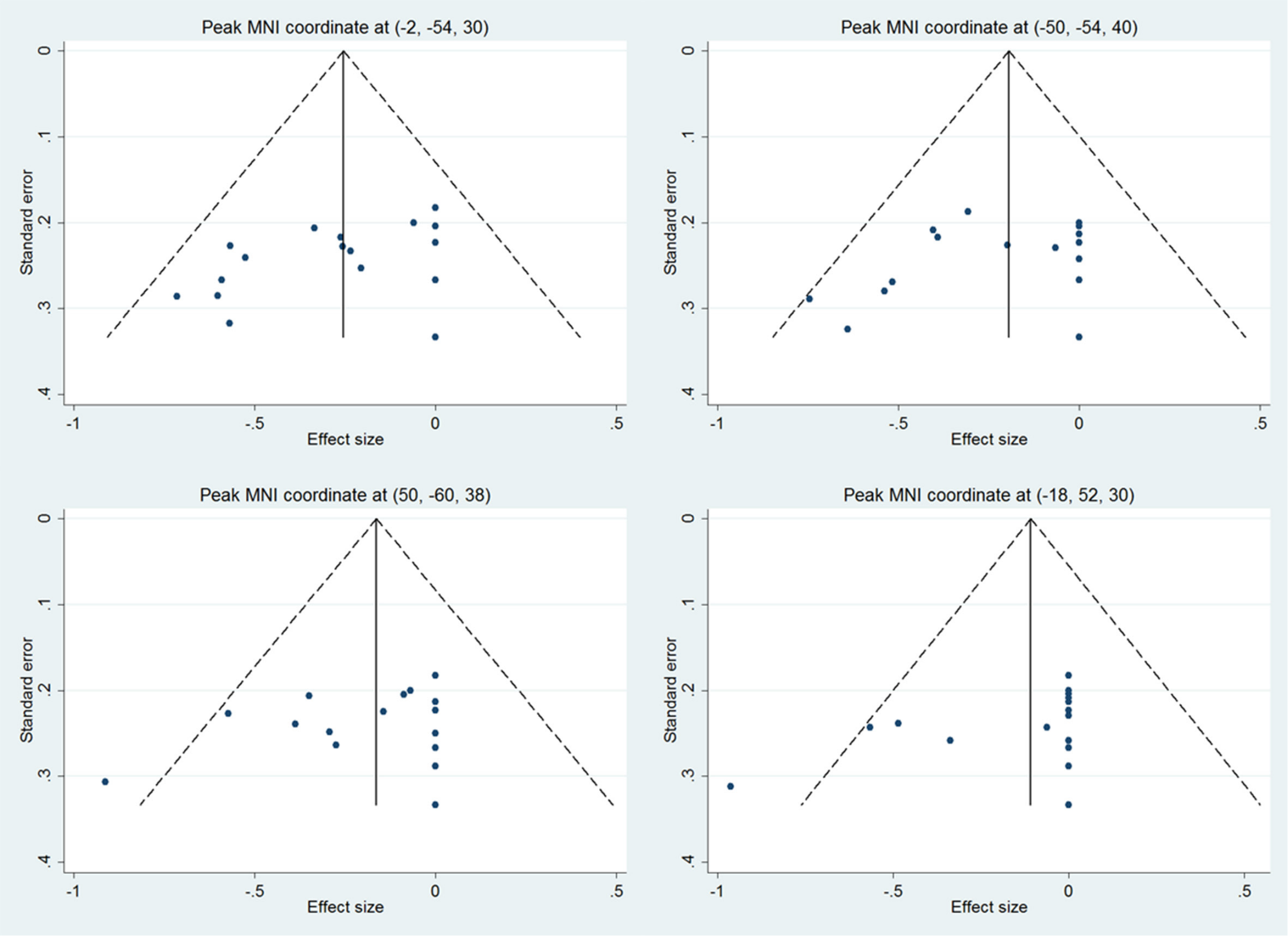

Supplementary Figure 1: Funnel plots of the peak coordinates from the main meta-analysis for detecting publication bias. Abbreviations: MNI, Montreal Neurological Institute

Supplementary Table 1: Diagnostic criteria and clinical assessment for AD. See_Supplementary_Table 1

Supplementary Table 2: Quality assessment checklist

Category 1: Subjects	Score (0/0.5/1)#
1 Patients were evaluated prospectively, specific diagnostic criteria were applied, and demographic data was reported	
2 Healthy comparison subjects were evaluated prospectively, psychiatric and medical illnesses were excluded	
3 Important variables (e.g. age, gender, severity of illness) were checked, either by stratification or statistically	
4 Sample size per group > 10	
**Category 2: Methods for image acquisition and analysis**	
5 Whole brain analysis was automated with no a priori regional selection	
6 Coordinates reported in a standard space	
7 The imaging technique used was clearly described so that it could be reproduced	
8 Measurements were clearly described so that they could be reproduced	
**Category 3: Results and conclusions**	
9 Statistical parameters were provided for significant, and important non-significant, differences	
10 Conclusions were consistent with the results obtained and the limitations were discussed	
	**TOTAL /10**

Abbreviations: #, 1 point per criterion fully satisfied, 0.5 for partially satisfied, 0 for not satisfied.
